# Evaluation of monitoring critical ill children with traumatic brain injury

**DOI:** 10.2478/jccm-2025-0001

**Published:** 2025-01-31

**Authors:** Merve Misirlioglu, Dincer Yildizdas, Faruk Ekinci, Ozden Ozgur Horoz, Gulen Gul Mert

**Affiliations:** Department of Pediatric Intensive Care, Mersin University Faculty of Medicine, Mersin, Türkiye; Department of Pediatric Intensive Care, Cukurova University Faculty of Medicine, Adana, Türkiye; Department of Pediatric Neurology, Cukurova University Faculty of Medicine, Adana, Türkiye

**Keywords:** child, intensive care, monitoring, traumatic brain injury

## Abstract

**Introduction:**

In traumatic brain injury (TBI), direct information can be obtained about cerebral blood flow, brain tissue oxygenation and cerebral perfusion pressure values. More importantly, an idea about the changes in these measurements can be obtained with multidimensional monitoring and widely used monitoring methods.

**Aim of the study:**

We aimed to evaluate the monitoring of critically ill children who were followed up in our pediatric intensive care unit (PICU) due to TBI.

**Material and Method:**

Twenty-eight patients with head trauma who were followed up in our tertiary PICU between 2018 and 2020 were included in the study. Cerebral tissue oxygenation, optic nerve sheath diameter (ONSD), Glasgow coma score (GCS) and Glasgow Outcome Score (GOSE) values were obtained from retrospective file records and examined.

**Results:**

Male gender was 71.4% (n=20). When we classified TBI according to GCS, 50% (n=14) had moderate TBI and 50% had severe TBI. On the first day in the poor prognosis group, ONSD and nICP were found to be higher than in the good prognosis group (for ONSD, p=0.01; and for nICP, p=0.004). On the second day of hospitalization, the ONSD and nICP were significantly higher in the poor prognosis group than in the good prognosis group (for ONSD p=0.002; and for nICP p= 0.001). Cerebral tissue oxygenation values measured on the first and second days decreased significantly on the second day in both the good and poor prognosis groups (p=0.03, 0.006). In the good prognosis group, a statistically significant decrease was found in ONSD and nICP measurements taken on the 2nd day compared to the measurements taken at the time of hospitalization (for ONSD p=0.004; for nICP p<0.001).

**Conclusion:**

The aim of multidimensional follow-up in traumatic brain injury is to protect the brain from both primary and secondary damage; for this reason, it should be followed closely with multimonitoring methods that are possibly multidisciplinary.

## Introduction

Traumas are still a cause of morbidity and mortality in the childhood age group [1]. According to the data of the Turkish Statistical Institute (TSI), by the end of 2022, 26.5% of Turkey's population consisted of children [2]. According to TSI death and cause of death statistics, the highest number of child deaths in 2021 occurred due to poisoning and external injuries, namely, traumas [3]. The risk of bleeding is high in children because the blood supply to the scalp is higher than in adults. Low-volume bleeding can lead to rapid deterioration of hemodynamic and sequelae in children. The higher head-to-body ratio in the pediatric age group compared to adults, thinner bone layers in the skull and incomplete preservation of the intracranial structures cause a more severe course of head trauma in children [4,5].

Traumatic brain injury (TBI) includes intracranial hemorrhage, pneumocephalus, brain edema, collapsed skull fracture or skull diastasis occurs after trauma. Clinically significant TBI is the need for neurosurgical intervention, intubation for at least 24 hours, hospitalization for two or more days due to TBI, or TBI-related death [6,7]. TBI is classified as primary and secondary injury. While primary damage is almost entirely caused by trauma, secondary injury occur due to conditions such as hypoxia, hypotension, hypercarbia and anemia. Seizures or increased intracranial pressure may occur due to TBI [8].

Patients with TBI and a Glasgow coma score (GCS) of 8 or below are monitored for increased intracranial pressure (ICP) and are followed up to maintain adequate cerebral perfusion pressure (CPP). While monitoring the damaged brain, it is necessary to detect the physiological events that may cause secondary injury before they cause irreversible damage and, if possible, to give the necessary treatment earlier. The main purpose of monitoring is to determine whether the CPP is at an adequate level. To achieve this, it is necessary to provide information about cerebral blood flow, brain tissue oxygenation and CPP values [4,5,6,7,8]. In our study, we aimed to evaluate the monitoring methods of critically ill children who were followed up due to traumatic brain injury.

## Material And Methods

Twenty-eight patients with head trauma who were followed up in our tertiary pediatric intensive care unit (PICU) between 2018 and 2020 years were included in the study. Pediatric trauma score (PTS), GCS and Glasgow outcome score-extended (GOSE) values, optic nerve sheath diameters (ONSD), cerebral near infrared spectroscopy (NIRS) values were obtained from the records in the retrospective patient files.

Pediatric trauma score, GCS and GOSE values were recorded to compare the severity of injury, to predict disease prognosis and to determine the risk of morbidity in trauma patients. The GCS is used to assess the level of consciousness in both adults and children. It assesses eye, motor and verbal responses, assigning points. The GCS total score can range between 3 (lowest score) and 15 (highest score) [9,10]. To evaluate the consciousness levels of the patients, GCS values were recorded each day of follow-up and head traumas were grouped. Mild TBI was classified as GCS 14–15, moderate TBI as GCS 9–13, and severe TBI as GCS ≤8. The PTS is assessed by evaluating the patient's airway patency, consciousness status, body weight, systolic blood pressure, presence of open wounds and roughly the presence of skeletal trauma. The total score ranges from −6 to +12, with a score below eight points indicating potentially significant trauma. PTS is an important scoring system in predicting patient triage [11,12]. GOSE was evaluated at the discharge of the patients. It is used to standardize the level of recovery and the measurement of functionality in the process following brain injury. Measurements are based on the practitioner's observation and evaluation, with 1:death, 2:vegetative stat (minimal responsiveness), 3-4:severe disability, 5-6:moderate disability, 7:recovered but very mild neurological or psychological disability, and 8:fully healed [13]. The GOSE values ranging from1 to 6 were classified as poor prognosis and 7 to 8 as good prognosis.

**Fig. 1.**
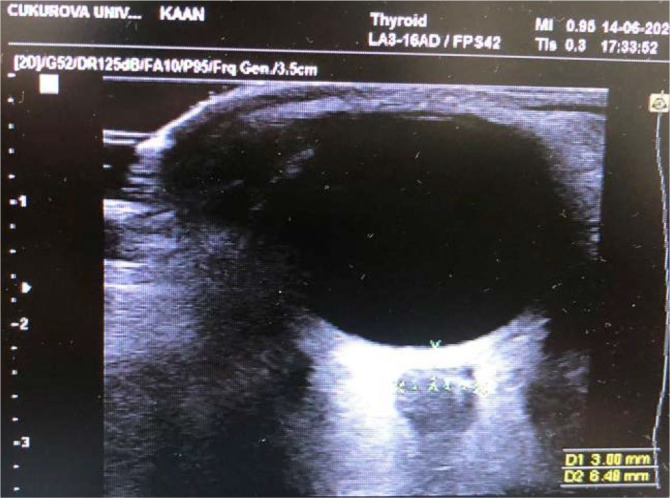
Measurement of optic nerve sheath diameter of our patient by ultrasonography

Optic nerve sheath diameter (ONSD) measurement was performed by ultrasonography (US), a noninvasive method, for detecting and monitoring the presence of increased intracranial pressure in patients with TBI [14]. The ONSD measurements were made by an experienced clinician who had received training on the subject (Figure 1). Ultrasound-based non-invasive methods are gaining interest and have shown promising results. However, there are also studies in which these formulas are used in PICU, and we aimed to use these formulas. After obtaining ONSD measurements, noninvasive intracranial pressures (nICP) were calculated. Non-invasive ICP derived from ONSD was estimated according to formula: “nICP_ONSD_ = 5x ONSD−13.92 (mm Hg)”[15,16]. Cerebral perfusion pressure (CPP) was the pressure gradient that will provide cerebral blood flow. It was calculated by subtracting the intracranial pressure from the mean arterial pressure. In addition to vital signs follow-ups in our PICU, we follow PTS, GCS, GOSE, ONSD, nICP and CPP in every patient with head trauma. These evaluations are made by pediatric intensive care specialists and routinely recorded in patient files. Our patients' NIRS measurements, ONSD and noninvasive ICP values were recorded and evaluated 24 hours (on the first day) and 48 hours (on the second day) after admission to the PICU. Regional tissue oxygenation was recorded by monitoring with NIRS, which is used noninvasively. Following skin cleansing, self-adhesive probes were used. For cerebral measurement, pediatric probes placed in the right and left frontal regions were connected to the NIRS device to monitor cerebral oxygenation.

The study was ethically approved by the Cukurova University Non-Interventional Clinical Research Ethics Committee (Approval No:33/19, Date: May 5, 2023). Because of retrospective research, family consents' were not required.

### Statistical Analysis

All statistical analyses were performed with IBM SPSS (Version 20.0) Statistics Package Program. The distribution of variables was investigated using visual (histogram and probability plots) and analytical methods (Kolmogorov Smirnov/Shapiro-Wilk's test) to determine whether they are normally distributed or not. Descriptive analyses of continuous variables were presented using mean ± standard deviations (SD) for normally distributed variables, whereas median and interquartile range (IQR) values were used for non-normally distributed and ordinal variables. Number and percentage values were used for descriptive data of categorical variables. The student's t-test was used to compare parameters between Good GOSE and Poor GOSE. Paired Student's t-test was used to compare the measurements at different time points (admission, first day, and second day) for NIRS, ONSD, nICP, and CPP measurements. A value of p < 0.05 was accepted as the level of statistical significance.

## Results

In our PICU, 71.4% (n=20) of 28 patients followed due to TBI were male. Patients' ages were 83.14 ± 65.53 (min 7, max 204) months and their length of stay in the PICU were 12.9 ± 6.6 (min 4, max 27) days. The PTS values were 3.2 ± 4.0 (min −6, max 8). The most common cause of trauma was out-of-vehicle traffic accidents (42.9%, n=12), which was followed by falls from a height (21.4%, n=6), blunt trauma (17.9%, n=5), in-vehicle traffic accidents (14.3%, n=4) and other reasons (3.6%, n=1). When we examined the type of TBI, 53.6% (n=15) of extraparenchymal injuries included epidural and subdural hemorrhage, subarachnoid hemorrhages, and intraventricular hemorrhage; while 35.7% (n=10) of intraparenchymal injuries included intracerebral hemorrhage and diffuse axonal injury, and in 10.7% (n=3) bone fractures were present. Other organ/system injuries were also present in 57.1% (n=16) of our patients. There was a history of seizures after trauma in 82.1% (n=23). Patients' characteristics are shown in Table 1.

**Table 1.** Patients' characteristics

**Variable**	**Value**
*Cause of trauma*	
Out-of-vehicle traffic accidents	42.9 % (n=12)
Falls from a height	21.4 % (n=6)
Blunt trauma	17.9 % (n=5)
In-vehicle traffic accidents	14.3 % (n=4)
Other reasons	3.6 % (n=1)

*The type of traumatic brain injury*	
Extraparenchymal injuries	53.6 % (n=15)
Intraparenchymal injuries	35.7% (n=10)
Bone fractures	10.7% (n=3)

*The treatments*	
Neurosurgery	25% (n=7)
Antibiotic therapy	96.4% (n=27)
Antiepileptic drugs	92.9% (n=26)
Anti-edema treatment	96.4% (n=27)
Thiopental infusion	3.6% (n=1)
Decompressive craniotomy procedure	3.6% (n=1)
Normothermia	100% (n=28)

In terms of the treatments they received, 25% (n=7) had undergone neurosurgery, 96.4% (n=27) had received antibiotic therapy, 92.9% (n=26) had started antiepileptic drugs, and 96.4% had started anti-edema treatment. While thiopental infusion was administered in 3.6% (n=1), a decompressive craniotomy procedure was performed in 1 critically ill child (3.6%) (table 1). None of them received steroid as an anti-edema treatment. Normothermia was administered to all cases, but hypothermia was not administered. For respiratory support application, 50% (n=14) of patients were followed up on invasive mechanical ventilation. Sedation analgesia was administered to 46.4% (n=13) of patients.

**Fig. 2.**
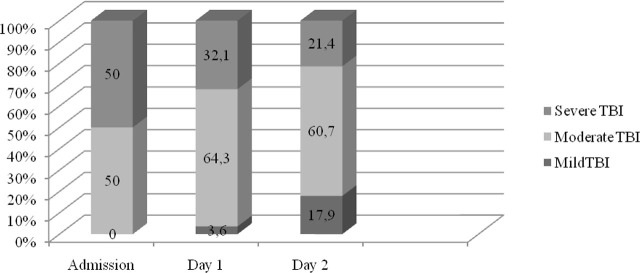
The change of traumatic brain injury classification according to the Glasgow coma score of the patients by days (%)

**Fig. 3.**
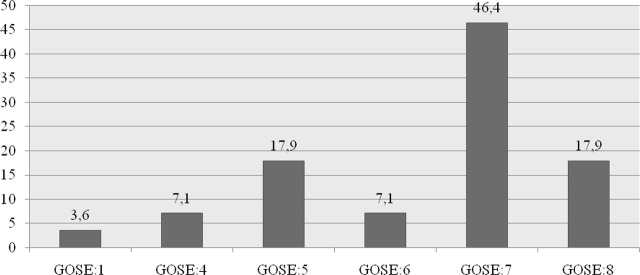
Evaluation of patients to Glasgow Outcome Scores-E

The GCS score at admission to PICU ranged from 5 to 13, with 50% classified as moderate and the other 50% classified as severe TBI. Evaluation of follow-up findings of patients in the PICU according to days is shown in Table 2. ONSD measurements could not be performed in 4 of our patients due to orbital damage; ONSD and noninvasive ICP results of 24 patients were evaluated. Mortality occurred in only one patient (3.6%); factors affecting mortality could not be examined because our mortality rate was not sufficient to make further analyses. TBI according to GCS as a result of daily evaluation is shown in Figure 2. Their evaluation according to the GOSE at discharge from the hospital is shown in Figure 3.

**Table 2.** Evaluation of the monitoring findings of the patients in the pediatric intensive care unit by days

	**PICU admission** **Mean ± SD** **Min - Max**	**Hospitalization Day1** **Mean ± SD** **Min - Max**	**Hospitalization Day2** **Mean ± SD** **Min - Max**
Mean Arterial Pressure (mmHg)	82.3 ± 16.8	79.6 ± 13.2	79.5 ± 10.7
	58–120	62–109	63–108

Glasgow Coma Scores	8.8 ± 2.3	9.8 ± 2.3	11.0 ± 2.5
	5–13	6–14	5–14

Optic Nerve Sheath Diameters (mm) n=24)	5.8 ± 0.4	5.8 ± 0.5	5.7 ± 0.5
	4.7–6.7	4.6–6.7	4.6–7.0

NIRS (%)	74.3 ± 11.9	74.2 ± 9.9	70.4 ± 8.2
	50–91	55–95	55–89

Noninvasive ICP (mmHg)	16.1 ±1.9	15.9 ± 2.1	15.0 ± 2.5
	11.5–20.6	11–21	11–21

Cerebral Perfusion Pressure (mmHg)	67.8 ± 16.7	64.3 ± 11.9	63.8 ± 7.8
	40–102	46–88	50–80

NIRS: Near Infrared Spectroscopy, ICP: Intracranial pressure, PICU: Pediatric Intensive Care Unit, mmHg: Millimeter of Hg, Hg: Chemical symbol of mercury, mm: millimeter.

Optic nerve sheath diameter, noninvasive ICP, CPP, NIRS values of critically ill pediatric patients with TBI during the first 72 hours in the PICU and GOSE values at hospital discharge were recorded and the relationships between these data were examined. The GOSE values ranging from 1 to 6 were classified as poor prognosis and 7 to 8 as good prognosis. Table 3 was showed the comparison of the data on the day of admission to the PICU, day 1 and day 2 between patients with good and poor prognosis. There was no significant difference in monitoring data (NIRS, ONSD, nICP and CPP) between the two groups divided according to GOSE on the day of admission to the PICU (p>0.05). When the data on the first day of hospitalization were examined, there was a statistically significant difference between the two groups in the ONSD and nICP data. In the poor prognosis group, ONSD and nICP were found to be higher than in the good prognosis group (for ONSD p=0.01; and for nICP p=0.004). When the data on the second day of hospitalization were examined, ONSD and nICP were significantly higher in the group with poor prognosis than in the group with good prognosis (for ONSD p=0.002; and for nICP p= 0.001)(Table 3). Regarding the changes in our data according to the days, the NIRS values measured on the first and second days decreased significantly on the second day in both the good and poor prognosis groups (p=0.03, 0.006). In the good prognosis group, a statistically significant decrease was found in ONSD and nICP measurements taken on the second day compared to the measurements taken at the time of hospitalization (for ONSD p=0.004; for nICP p<0.001) (Table 4).

**Table 3.** Evaluations of the prognosis according to the follow-up variables according to the days of hospitalization in the pediatric intensive care unit

**Time**	**Variable**	**Prognosis**	**Mean ± SD**	**p value**
PICU admission	NIRS	Good(GOSE 7-8) (n=18)	73.6±10.8	0.71
		Poor (GOSE 1-6)(n=10)	75.4±14.1	

	ONSD	Good (GOSE 7-8)(n=16)	5.7±0.4	0.19
		Poor (GOSE 1-6)(n=8)	5.9±0.5	

	nICP	Good (GOSE 7-8) (n=16)	15.7±1.8	0.23
		Poor (GOSE 1-6) (n=8)	16.7±2.3	

	CPP	Good (GOSE 7-8) (n=16)	64.6±14.1	0.20
		Poor (GOSE 1-6) (n=8)	74.0±20.6	

Hospitalization Day1	NIRS	Good (GOSE 7-8)	72.7±10.3	0.28
		Poor (GOSE 1-6)	76.9±9.0	

	ONSD	Good (GOSE 7-8)	5.7±0.5	0.01^*^
		Poor (GOSE 1-6)	6.1±0.4	

	nICP	Good (GOSE 7-8)	15.1±1.8	0.004^*^
		Poor (GOSE 1-6)	17.6±1.1	

	CPP	Good (GOSE 7-8)	64.7±11.6	0.81
		Poor (GOSE 1-6)	63.4±13.5	

Hospitalization Day2	NIRS	Good (GOSE 7-8)	69.1±8.0	0.29
		Poor (GOSE 1-6)	72.6±8.5	

	ONSD	Good (GOSE 7-8)	5.5±0.4	0.002^*^
		Poor (GOSE 1-6)	6.1±0.5	

	nICP	Good (GOSE 7-8)	13.9±1.8	0.001^*^
		Poor (GOSE 1-6)	17.2±2.3	

	CPP	Good (GOSE 7-8)	62.9±5.7	0.43
		Poor (GOSE 1-6)	65.6±11.1	

* t-test; NIRS: Near Infrared Spectroscopy, ONSD: Optic Nerve Sheath Diameter, nICP: Noninvasive Intracranial Pressure, CPP: Cerebral Perfusion Pressure, GOSE: Glasgow Outcome Scale-Extended, PICU: Pediatric Intensive Care Unit.

**Table 4.** Comparison of prognosis according to neuromonitorization parameters between days

	p value^*^	**GOSE 7-8 (Good prognosis)**	**GOSE 1-6 (Poor Prognosis)**	p value ^*^
Admission NIRS		75.4±10.0	76.6±11.2	0.572
Day 1 NIRS	0.473	73.7±10.4	78.0±7.4	

Admission NIRS		75.4±10.0	76.6±11.2	0.360
Day 2 NIRS	0.009	69.7±8.2	74.1±8.3	

Day 1 NIRS		73.7±10.4	78.0±7.4	0.006
Day 2 NIRS	0.003	69.7±8.2	74.1±8.3	

Admission ONSD		5.7±0.4	6.0±0.4	0.203
Day 1 ONSD	0.534	5.7±0.5	6.1±0.4	

Admission ONSD		5.7±0.4	6.0±0.4	0.449
Day 2 ONSD	0.004	5.5±0.4	6.1±0.5	

Day 1 ONSD		5.7±0.5	6.1±0.4	1.000
Day 2 ONSD	0.140	5.5±0.4	6.1±0.5	

Admission nICP		15.7±1.8	16.7±2.3	0.243
Day 1 nICP	0.097	15.1±1.8	17.6±1.7	

Admission nICP		15.7±1.8	16.7±2.3	0.729
Day 2 nICP	<0.001	14.0±1.8	17.2±2.3	

Day 1 nICP		15.1±1.8	17.6±1.7	0.535
Day 2 nICP	0.002	14.0±1.8	17.2±2.3	

Admission CPP		64.6±14.1	74.0±20.6	0.240
Day 1 CPP	0.987	64.7±11.6	63.4±13.5	

Admission CPP		64.6±14.1	74.0±20.6	0.128
Day 2 CPP	0.604	62.9±5.7	65.6±11.1	

Day 1 CPP		64.7±11.6	63.4±13.5	0.687
Day 2 CPP	0.481	62.9±5.7	65.6±11.1	

* *p: paired samples t test*; NIRS: Near Infrared Spectroscopy, ONSD: Optic Nerve Sheath Diameter, nICP: Noninvasive Intracranial Pressure, CPP: Cerebral Perfusion Pressure, GOSE: Glasgow Outcome Scale-Extended.

## Discussion

Twenty-eight patients with head trauma who were followed up in our tertiary pediatric intensive care unit between 2018 and 2020 years were included in the study. In the first 72 hours of critically ill children with TBI in the PICU, advanced neuromonitoring parameters such as ONSD, noninvasive ICP, CPP, and NIRS values were recorded. The GOSE values between 1 and 6 were classified as poor prognosis and 7 to 8 as good prognosis. The ONSD and nICP were higher in the group with poor prognosis than in the group with good prognosis. When the changes in our data according to the days were examined, the NIRS values measured on the first and second days decreased statistically on the second day in both the good and bad prognosis groups. In the good prognosis group, ONSD and nICP were found to decrease on the second day compared to the values at admission to the PICU.

Traumatic brain injury is the most common form of pediatric trauma and also the most common cause of trauma-related death and morbidity [17]. Providing ventilation and oxygenation in childhood head traumas, regulating fluid and electrolyte balance, optimizing cerebral metabolism, preventing herniation and secondary cerebral tissue damage as a result of increased intracranial pressure constitute the basis of TBI follow-up and treatment strategies [18]. In addition to vital signs follow-ups in our PICU, we follow PTS, GCS, GOSE, ONSD, nICP and CPP in every patient with head trauma. The GCS is used to assess the level of consciousness. The GCS total score can range between 3 (lowest score) and 15 (highest score) [9,10]. Mild TBI was classified as GCS 14–15, moderate TBI as GCS 9–13, and severe TBI as GCS ≤8. Patients with mild head trauma are followed up in our hospital; according to our results, it has been determined that pediatric patients with moderate and severe head trauma are followed up in our PICU. The PTS ranges from −6 to +12, with a total score below 8 indicating potentially significant trauma. It is an important scoring system in predicting patient triage and mortality [11,12]. The PTSs of our patients ranged from −6 to +8 and potentially significant trauma patients were admitted to the PICU. The GOSE is used to standardize the measurement of recovery and functionality in the process following the brain injury [13]. While 46.4% of our children had lower good recovery, 17.9% were evaluated as having upper good recovery. The majority (64.3%) comprised the group with good prognosis (GOSE 7-8). When the patients with severe head trauma in adults were evaluated based on the GOSE during a 4-year period, the GOSE was considered a group with a good prognosis of 4 and above, reaching to 12% at the end of two weeks and 45% at the end of three months. Although the recovery of traumas in the adult age group is a longer and more difficult process, it shows higher morbidity and impairment [19].

In the follow-up of patients, the use of noninvasive methods is becoming increasingly common due to the difficulty, troublesome nature, cost and complications of invasive methods. The ONSD measurement is performed by US, which is a noninvasive method for detecting and monitoring the presence of increased ICP in patients with TBI [20]. It is performed by obtaining an image from the area between the dural sheaths in hyper echoic appearance located at the edge of the hypo echoic subarachnoid area surrounding the optic nerve. ONSD values < 4 mm in children ≤ 1 year and values < 4.5 mm in children > 1 year are assumed as normal, values above 5 mm in children > 4 years are assumed as abnormal [21,22,23]. In the study cohort; our patients' ONSD mean values were 5.8 ± 0.4 on admission to the intensive care unit; ONSD values were similar on the first and second days.

Noninvasive ICP are calculated after the ONSD measurements are obtained. There were also studies in which these formulas were used in adult and pediatric intensive care units. Robba et al. studied 10 patients under the age of 16 who had an indication for invasive intracranial pressure measurement. The aim of this study was to assess the relationship between ICP and different ultrasound–based methods in neurocritical care pediatric patients. This study analysed 107 measurements from 10 paediatric patients. Non-invasive ICP derived from ONSD was estimated according to the regression analysis between ICP and ONSD obtained from our previous study in a cohort of adult TBI patients [16]. Non-invasive ICP derived from ONSD was estimated according to formula: “nICP_ONSD_= 5x ONSD−13.92 (mm Hg)”. Results from linear regression demonstrated that, among the nICP methods, ONSD had the best correlation with ICP (r = 0.852 (p < 0.0001)) [15]. In cases with traumatic brain injury, monitoring with ICP measurements is recommended; ICP varies according to age, but is recommended to be kept below 20 mmHg [18]. Non invasive ICP values in our study were between 11 mm Hg and 21 mm Hg in the study cohort. Based on the data obtained in our study, ONSD and nICP were higher in the group with poor prognosis than in the group with good prognosis. In the good prognosis group, ONSD and nICP were found to decrease on the 2nd day compared to the values at admission to the pediatric intensive care unit.

Near-infrared spectroscopy measures regional oxygenation by interpreting oxy- and deoxyhemoglobin signals. The NIRS has become a routine part of our clinical practice for monitoring cerebral and somatic oxygenation in critically ill patients [24]. There is no clear information regarding the distribution of pediatric NIRS values for different tissue beds in the literature. Generally, the range of rSO2 is 50 to 85% [25]. In the NIRS follow-up of the patients in our study, it ranged from 50 to 91. Regarding the changes in our data according to the days, the NIRS values measured on the first and second days decreased statistically on the second day in both the good and bad prognosis groups.

In the follow-up with ICP measurements in cases with TBI, although ICP varies by age, the cerebral per-fusion pressure should be kept below 20 mm Hg and above 40 mm Hg [18,26]. Although ONSD and ICP values increase due to brain edema on the first day, these values decrease due to the absence of worsening edema in the follow-up. We think that no statistically significant difference was detected because the cerebral perfusion pressures of our patients were kept at target values according to ICP. As the severity of brain damage in our patients increased, intracranial pressure increased, resulting in increased ONSD and ICP, which were found to be associated with poor prognosis. In our study; the ONSD and nICP were significantly higher in the poor prognosis group than in the good prognosis group. In a retrospective analysis of 56 pediatric patients with traumatic brain injury, increasing ICP and decreasing CPP were found to be associated with poor prognosis [27]. The pediatric guideline recommended a target of 40 mmHg [18]. A recent study was suggested CPP thresholds of more than 60 mmHg in adults, 50 to 60 mmHg in children 6 to 17 years of age, and more than 40 mmHg in children aged <5 years [28]. In our cases, CPPs were over 40 mmHg.

Multimodal advanced neuromonitoring also includes methods such as jugular venous oximetry, brain tissue oxygen tension monitoring (PbtO2), cerebral micro dialysis and thermal diffusion flowmetry [29]. Since these methods are not available in our center, they cannot be used for patient evaluation and are rare in hospitals.

The limitations of our study are the low number of patients, the low mortality rate, the inability to analyze the factors affecting mortality, the inability to compare noninvasive and invasive ICP measurements due to the inability to insert an invasive ICP monitoring catheter, and the lack of transcranial Doppler measurements. Our study was conducted retrospectively, and ICP values were obtained by calculating noninvasively measured ONSD values. Prospective studies with a large number of patients are needed to address this issue.

## Conclusions

Severe TBI is often complicated by cerebral hypoxia, hypoperfusion, and edema, leading to secondary neurologic injury and worse outcome. The aim of multidimensional follow-up in traumatic brain injury is to protect the brain from both primary and secondary damage. In our study, we aimed to evaluate the monitoring methods of critically ill children who were followed up in our PICU due to TBI.
